# Swāsthya, an integrated chronic condition management programme for families of patients with hypertension and diabetes mellitus: a study protocol for a randomised controlled trial

**DOI:** 10.1186/s12875-020-01357-w

**Published:** 2021-01-09

**Authors:** M D Saju, Bindiya M Varghese, Lorane Scaria, Anuja Maria Benny, Shilpa V Yohannan, Natania Cheguvera, S P Rajeev, Amuthavalli Thiyagarajan Jotheeswaran

**Affiliations:** 1 Department of Social Work, Rajagiri International Centre for Consortium Research in Social Care, Kalamassery, Kochi, Kerala India; 2 Rajagiri College of Social Sciences (Autonomous), Rajagiri P.O, Kalamassery, Kochi, Kerala 683 104 India; 3 Department of Computer Science, Rajagiri College of Social Sciences (Autonomous), Kalamassery, Kochi, Kerala India; 4 Rajagiri International Centre for Consortium Research in Social Care, Kalamassery, Kochi, Kerala India; 5 Department of Social Work, Rajagiri College of Social Sciences (Autonomous), Kalamassery, Kochi, Kerala India; 6 Department of Ageing and Life Course, WHO, Geneva, GE Switzerland; 7 Institute of Psychiatry, Psychology and Neurosciences, King’s College London, London, UK

**Keywords:** Hypertension, Diabetes mellitus, Integrated care model, Study protocol, Medical compliance, India

## Abstract

**Background:**

Kerala is known as the diabetes mellitus (DM) and hypertension (HTN) capital of the world, thus compelling health professionals to model strategies, addressing their social, behavioural, and cognitive risk factors and eliminating various barriers to management. This paper describes the protocol of our study that aims to examine the effectiveness and sustainability of an integrated care model for the management of chronic conditions and their risk factors through a family-based intervention. The proposed care model targets to modify systems and processes that predispose to chronic conditions by enhancing social cohesion and social networks, preventing lifestyle risks, developing iterative cognitive interventions, and engaging the family into customised treatment adherence strategies navigated by community health social workers (CHSWs).

**Methods:**

A cluster randomised controlled trial (RCT) in selected participants will be conducted involving additional assessments prior to the baseline assessment. The assessment will identify and categorise patients into four risk groups, namely behavioural, social, cognitive, and multiple, based on dominant risks identified. Eligible participants will be randomly allocated (at a ratio of 1:1) into the intervention or control arm. The intervention arm will receive social, behavioural, and cognitive or multiple interventions corresponding to the identified risk groups, whereas the control arm will receive general intervention. Both the groups will be followed up at 6 months and 12 months post baseline to measure outcomes. The primary outcome will be the control of HTN and DM, and secondary outcomes include decreased depression and anxiety and improved functioning, social cohesion, and social network linkages. The sustainability and scalability of this intervention will be assessed through cost effectiveness, acceptability, and user friendliness of the integrated approach by performing a qualitative evaluation.

**Discussion:**

This RCT will inform the potential paradigm shift from a medical model of chronic condition management to a multidimensional, multisystem, and multidisciplinary convergence model navigated by CHSWs. Such a model is not currently considered in the management of chronic conditions in Kerala.

**Trial registration:**

Trial has been prospectively registered on Clinical Trial Registry of India- CTRI/2020/12/029474 on 1st December 2020.

## Background

The poor control status of patients [1] resulting from factors such as a lack of an integrated healthcare system and poor adherence to prescribed treatment recommendations and medications [2] is the key reason for an increase in non-communicable diseases (NCDs). Among these factors, treatment adherence and the management of behavioural or lifestyle risk factors, such as smoking and tobacco use, sedentary lifestyle, poor diet, and alcohol abuse [3], are potentially amenable. Various social risk factors such as poverty [4], a lack of social inclusion and support [5], low social class, low income, and low education levels [6] are other barriers to treatment adherence and behavioural risks. In addition, the non-diagnosis of a chronic condition due to a lack of awareness and deficient healthcare access [7] can be considered a social risk factor.

Patients’ poor control status demonstrates the urgency in directing our interventional focus on treatment adherence [8]. Recent studies have found that social and behavioural risk factors predispose to non-adherence to treatment [9, 10].

The name of our intervention programme is Swāsthya, which is a unique concept from Sanskrit denoting a holistic and integrated approach to the health and well-being of the body, mind, and soul. Swāsthya is an integrated chronic condition management programme based on cross-sectional data collected from a semi-urban community on various behavioural, social, and cognitive risks of chronic conditions, namely diabetes mellitus (DM) and hypertension (HTN). This intervention model specifically targets social factors that are amenable to modification by healthcare practitioners working within the existing health system. We performed a rapid systematic review to critically explore existing models and modifiers. The absence of evidence highlights the need to develop a contextually relevant and theoretically driven intervention model for a community population. This intervention is modelled on the basis of the results of several phases of intervention development, namely scoping review [11], community survey [9], rapid systematic review, and qualitative interviews with patients with HTN or DM and health professionals in the community health ecosystem. This stakeholder-driven model explores behavioural, social, and cognitive factors clustered within individuals, families, and communities in Kerala.

The behavioural risk group was characterised by the following unhealthy habits and physical risk factors: unhealthy diet with a high level of sugar, oil, and salt intake; irregular and frequently erratic food patterns; a high level of alcohol consumption; smoking; and sedentary lifestyle.

The social risk group was characterised by inadequate family support, social isolation, financial distress, and a lack of meaningful social and interpersonal relationships. The social isolation of this group limits their access to health services and deprives them of healthy lifestyle educational resources. Financial distress is a significant but modifiable social risk factor that predicts noncompliance to treatment.

Stakeholders, particularly community health professionals, highlighted poor public awareness of the importance of adherence to medication and a lack of public awareness of government services, such as NCD clinics, as the most important barriers to treatment adherence. In addition, a misconception exists that the quality of privately purchased medications is higher than that of government-subsidised medications provided at primary health centres (PHCs).

Health professionals, particularly PHC doctors, suggested task shifting as the potential method to improve the management of risk factors for cardiovascular disease (CVD)in an integrated service delivery ecosystem, especially in the context of overcrowding and rushed consultations at PHCs. Moreover, they suggested that a care model should have a pyramidal platform wherein the system can categorise patients based on their care needs and establish a referral pathway to ensure the maximum coverage of services at a minimum cost; this type of model can be best suited for low- and middle-income countries (LMICs) such as India. In addition, the health professionals indicated that Accredited Social Health Activists (ASHAs), if trained, can deliver health education services including formal and informal resource linkage functions, social connectedness, and initial mental health screening.

Addressing the lack of family and other support for people with chronic diseases and providing psychological support are crucial to address the barriers to the management of risk factors for CVD. According to health care professionals, for a complex social intervention to be successful in India, it needs to account for resources in families and communities and its social context. Strengthening weak ties in people’s networks is crucial to connect them to health resources and public health communication.

This intervention aims to develop and implement a sustainable and cost-effective family-based [12] care package to address barriers to treatment adherence by utilising already existing human health resources. ASHAs, an untapped community-based health resource [13] are equipped to provide the care package based on the priorities of patients through task-shifting and task-sharing strategies. The uniqueness of this family-based, community-focused intervention model based on theory of change (ToC) is that community health social workers (CHSWs) play a key role to navigate change pathways by utilising their professional knowledge and competencies.

## Methods/design

### Aim of the study

The goal of this intervention is to improve treatment adherence and medical compliance by addressing social, behavioural, and cognitive barriers to ensure the control of chronic conditions such as HTN and DM. The objectives of the current intervention are as follows: 1) to allocate patients to various risk groups (behavioural, social, cognitive, and multiple) based on their exposure to dominant risk factors; 2) to implement a customised intervention programme specific to each risk group of patients to reduce their determined risk factors; 3) to assess changes in HTN and DM by making changes in behavioural factors, such as smoking, alcohol consumption, diet, and physical activity, social factors, such as social cohesion and social linkages with networks, and cognitive factors, such as reduction of depression and anxiety; and 4) to understand the feasibility and applicability of the ASHA-worker-delivered, social-worker-coordinated, integrated, health care model for the management of HTN and DM.

### Study design and setting

This is a two-arm cluster randomised controlled trial (RCT) designed to be executed in patients with DM or HTN in association with government-run PHCs and the existing public health ecosystem in Kerala, India. This study will be conducted in Ernakulam district, Kerala, India, with active support from University of York, United Kingdom, and University of Melbourne, Australia. The Ernakulam district is administratively divided into seven main revenue divisions, and Aluva is one of them [14]. The Aluva division is further divided into different units called panchayats (with approximately 25,000–30,000 people residing in each panchayat and each panchayat is further divided into different wards with approximately 1000 to 1500 people residing in each of them), and one panchayat from the Aluva division is selected for the intervention. PHCs and ASHA workers assigned to these geographical units will assist the execution of the intervention. ASHA workers are a network of community health workers who are often selected from the same community; thus, they have the acceptance and trust of the community people. A list of ASHA workers consenting to participate in this study will be prepared, and 10 of them will be randomly selected using computer generated random numbers for providing the intervention. Their assigned wards will be considered as intervention clusters for the current trial. A list of patients with DM or HTN from the selected wards will be obtained from the PHC registry through ASHA workers, and household interviews will be conducted for data collection. Three data collection time points are planned for both the arms: one at baseline and then after 6 months and 12 months after baseline.

Two levels of randomization will be employed in this study. Of the 10 ASHAs, 5 will be randomly allocated either to the treatment intervention arm (Swasthya intervention) or the usual care arm (no interventions other than detailed assessments). Computer-generated random numbers generated by an independent person who is not involved in the study will carry out the randomization of the ASHA workers to either group. Randomization of ASHAs and their clusters will be performed only after the collection of complete baseline data. Their assigned wards will be considered as intervention clusters for the current trial. To eliminate the possibility of contamination, these two groups of ASHA workers will be given training and orientation separately and each of them will be concealed by the group to which they belonged. Independent persons will do the outcome assessment and manage the data to ensure blinding to the Intervention content and its processes. The data analysts will receive the data only after locking the database.

### Participant recruitment and study procedure

Participants for the trial will be recruited according to eligibility criteria. Eligible families will be those with at least one family member with a confirmed diagnosis of HTN and/or DM. The medical records of the patient will be used to confirm the diagnosis. Informed consent will be obtained from all adult family members. In addition, family members must be either first-degree blood relatives or spouses of the patient. Bedridden and terminally ill patients will be excluded from the study. Participation will be completely voluntary and can be withdrawn at any point if they intent to.

#### Recruitment of participants

**Fig. 1:**
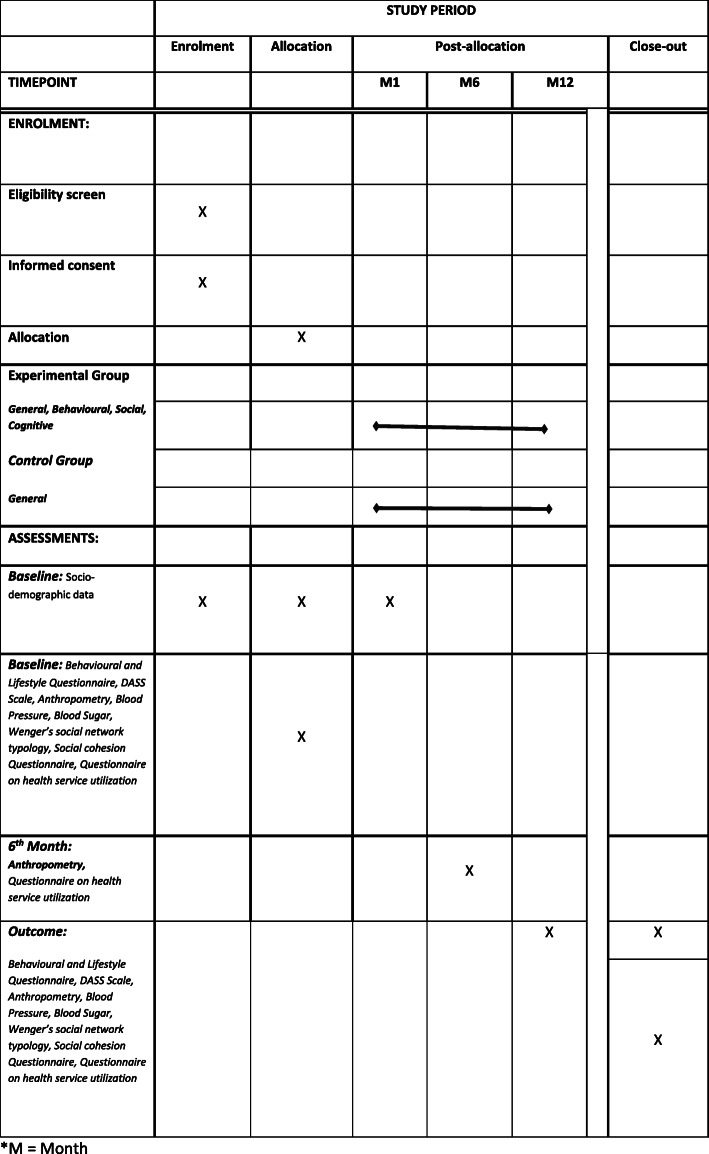
Assessment Schedule of Enrolment, Interventions and assessment

The recruitment of participants will be conducted in three stages as follows (Fig. 1).

##### First‐level assessment and recruitment

PHCs, with the help of ASHA workers, conduct annual surveys in their respective clusters to collect the information of people with chronic conditions. They collect information on sociodemographic factors such as house number, name, address, phone number, age, sex, type of house, socioeconomic status, education level, occupation, marital status, and receipt of pension, and health factors, such as whether a person has HTN, DM, high cholesterol, thyroid, any disability, or any other chronic condition. This list will be obtained from PHCs through ASHA workers for easy identification of the targeted population. The households of people with DM and/or HTN will be recruited for the next-level assessment. This first-level assessment will be supervised by CHSWs.

##### Second‐level assessment

ASHAs will perform the second-level assessment of the recruited participants. The second-level assessment consists of questions assessing various behavioural, social, and cognitive risks of participants. On the basis of major risk indicators determined through the assessment, CHSWs will categorise the participants into three risk groups, namely behavioural, social, and cognitive. Participants who fall into more than one risk group will be separately categorised into the multiple risk group.

##### Third‐level assessment

In the third-level assessment, CHSWs will accompany ASHA workers to the homes of these recruited patients for performing further detailed assessment. This assessment will comprise standardised tools that assess treatment adherence; medication compliance; and behavioural, social, and cognitive risk factors in patients with chronic illnesses. In addition, this assessment will identify the motivating and protective factors of participants to adhere to treatment and comply with medication. Moreover, DM, HTN, and body mass index (BMI) will be measured by junior public health nurses (JPHNs) in coordination with CHSWs.

#### Human resources for the assessment

ASHA workers will conduct the first-level (verbal screening) and second-level assessments (mobile App based), followed by the detailed baseline assessment performed by CHSWs. Along with ASHA workers, JPHNs will conduct clinical assessments including the measurement of blood pressure (BP) and blood sugar levels. The primary and secondary outcome measurements for determining the effectiveness of the intervention will be performed by a separate group of CHSWs in order to blind the hypothesis from the intervention team. The intervention will be functionally and geographically coordinated by CHSWs appointed in PHCs during the project implementation period.

### Study outcomes

Collection of data at baseline and follow-up at 6 months and 1 year will involve (a) the first-level assessment that includes a survey for collecting information regarding basic sociodemographic factors and verbal screening for examining chronic conditions in potential participants and (b) the second-level assessment that includes the use of standardised questionnaires to collect information on treatment adherence, medication compliance, behavioural risk factors (diet pattern, physical activity, tobacco, and alcohol consumption), social risk factors (perceived social support, neglect, and inclusion), and cognitive risk factors (depression, anxiety, stress), as well as a semi-structured interview schedule for identifying the motivating and protective factors of participants. In addition, trained staff will conduct BP, blood sugar level, and BMI measurements in all participants at three time points (baseline and 6 and 12 months after baseline). Anthropometric measurements will include height (in cm), weight (in kg), and waist circumference (in inches). BP and pulse rate will be measured using electronic BP monitors (Dr Morepen BP-02 Blood Pressure Monitor). Trained interviewers used a lancet for finger prick to obtain fresh capillary whole blood and a glucometer to measure the random glucose level. HTN will be measured twice in the sitting position at two time periods, and the average of the two readings will be used as the final value. Detailed survey measures are described in Table 1.

**Table 1:** Measurement domains and data collection time points

**Variables**	**Components**	**Measurement Tools**	**Baseline**	**6 months**	**12 months**
Sociodemographic variables		Age, sex, marital status, education, occupation, and family income	✔		
Behavioural measures	Tobacco use, Alcohol consumption, Diet Physical activity	Behavioural and Lifestyle Questionnaire	✔		✔
Cognitive measures	Depression Anxiety Stress	DASS scale	✔		✔
Clinical measures	Anthropometry	Waist circumference (measuring tape) Height (measuring tape) Weight (weighing machine)	✔	✔	✔
	Blood pressure	Dr Morepen Blood Pressure Calculator			
	Blood sugar	Glucometer (finger prick method)			
Social measures	Social networks	Wenger’s social network typologies	✔		✔
	Social cohesion	Social cohesion questionnaire			
Treatment adherence	Compliance to medications Adherence to Health check ups	Questionnaire on health service utilisation	✔	✔	✔
Programme evaluation	Group evaluation	Usefulness of group sessions, Utilisation of NGO services	4 month	8 month	12 month
	Other evaluation	Usage of health calendar, diary, etc.	4 month	8 month	12 month

#### Primary outcomes

Primary outcomes include an improvement in risk factor control status (BP < 140/90 mmHg and fasting blood glucose < 110 mg/dL) and abstinence from smoking or tobacco use.

#### Secondary outcomes

Secondary outcomes include optimal health indicators such as (a) increased compliance to medication as reported by family members; (b) a reduction in the quantity of salt, oil, sugar, and meat bought in a month; (c) an increased number of healthy food items prepared in the household in a month; (d) sticking to the diet chart and health calendar; (e) a reduction in or abstinence from alcohol consumption; (f) engagement in at least one physical activity; (g) increased engagement with family, friends, and church groups; (h) getting back to professional or academic life and attending work or school regularly; and (i) attainment of appropriate BMI(< 25 kg/m^2^). In addition, the outcomes included attending at least one group meeting, increased linkage with the community, formal and informal interactions with the community, utilisation of nongovernmental organisation (NGO) services, regularity in hospital visits, and decreased depression and anxiety.

### Intervention components

#### Usual care arm

The usual care arm will undergo the first- and second-level assessment. Patients with HTN and/or DM in the control arm will also be referred to PHCs or encouraged to access their preferred health care facility.

#### Treatment arm

We propose to utilise existing healthcare resources such as PHCs, ASHA workers, NGOs, and family counselling centres, which are already functioning in the community, for the intervention. The package comprising five separate modules on general, behavioural, social, cognitive, and multiple interventions prepared by the research team based on information collected through quantitative, qualitative, and rapid systematic review of existing evidence will be applied based on the risk categorisation.

General interventions will be delivered to all participants included in the intervention arm. Behavioural, social, and cognitive interventions will be separately administered to participants based on the group they belong to. For the fourth group, that is, the multiple risk groups, interventions will be customised based on their priority needs. All the interventions are developed targeting the entire family.

### Overview of intervention

The intervention is delivered to participants based on identified risk categories.

#### Intervention implementation, general

General intervention is administered to all participants in the intervention arm. General information comprises the importance of treatment adherence and adequate control of chronic conditions. An overview of intervention components is provided in Table 2.

**Table 2:** Overview of Interventions

**Participant Categories**	**Objectives**	**Interventions**	**Tools**	**Methods**
Behavioural risk group	To incorporate lifestyle modifications to manage diabetes and hypertension in patients	**Individual level** 1) Risk management strategies for alcohol consumption, smoking, sedentary lifestyle, and low-fat diet recipes, and specific monitoring strategies for salt, sugar, and oil intake. 2) Graded reduction strategies for alcohol consumption and smoking; Linking to de-addiction centres if necessary.	Healthy lifestyle- specific brochures, videos on anatomical explanation on unhealthy habits and its foreseeable effects, chronic condition-specific leaflets, and monitoring charts	Household visits by ASHA workers once in a month
		**Family level** 3) Obesity intervention programmes for potential patients by providing a customised diet plan.	Diet charts and recipe books	Information booklet-based family consultations by ASHA workers
		**Community level** 4) Community awareness generation regarding the effect of unhealthy habits and sedentary lifestyle on chronic conditions	Brochures, health calendars, and health-related videos	ASHA workers spend 10–15 minutes in community meetings to speak about healthy lifestyle
Social risk group	To bring about changes in individuals and families by promoting more social networks and ties with others in the community	**Family level** 1) Forming beneficiary groups of participants 2) Availing services such as free medicines, micro-pharmacy, doctor emergencies, and transportation facilities to hospitals	Brochures and handouts comprising details of already existing support systems	Individual- and family-level consultations and connecting to existing NGOs and other functionaries
		**Community level** 3) Awareness generation regarding support services, government welfare and health schemes, nongovernment organisations, and other informal provisions and resources	Beneficiary groups, and handouts with the details of agencies along with their address and phone number	Creation of beneficiary groups by ASHA workers, and distribution of brochures
Cognitive risk group	To modify the negative cognition of patients through cognitive techniques for better mental health	**Individual level** 1) Cognitive and behavioural steps, and referral to family counselling centres (FCCs)	Case management technique videos and awareness classes	FCC staff to provide therapy sessions, and ASHA workers to generate awareness on common mental health conditions
		**Family level** 2) Strategies to improve connections between family members 3) Community linkage for socially vulnerable patients	Increasing the frequency of visits to these vulnerable families	House visits by ASHA workers and encouraging frequent contacts

The major components of the intervention package include assessment, psychoeducation, risk-specific intervention, and linkage with emergency services.

### Intervention content

1. Brochure for health education with information regarding the importance of medication adherence
2. Instructions to families to monitor treatment adherence and medication compliance
3. Steps on how families can support (physically and emotionally) through reminders, positive encouragement, etc
4. Common strategies for behavioural risk management, such as the provision of a diet chart, healthy recipe book, health calendar, which is to be mounted on the kitchen wall, and pamphlets for simple and effective cognitive strategies such as relaxation techniques. ASHA workers will be responsible for implementing general interventions. They will explain in detail the aforementioned four strategies before handing out the brochures and pamphlets. The research team and CHSWs will perform monitoring and coordination during the intervention. One-day training will also be provided to all ASHA workers prior to the implementation of interventions.

In addition to the general intervention, participants in the treatment arm will be administered risk-group specific interventions as follows.

#### Intervention implementation: behavioural

Behavioural interventions are proposed for lifestyle modifications to manage DM and HTN and are widely used to gain control over chronic illnesses [15]. This intervention particularly focuses on bringing changes in alcohol consumption/dependence, smoking, physical inactivity/sedentary lifestyle, and improper food habits/uncontrolled diet. In addition, respondents will be linked to de-addiction centres if necessary.

#### Intervention implementation: social

Social interventions are proposed for improving treatment adherence, enhancing social cohesion, and improving social connections. Support from family and friends [16, 17], health centres [18], and peer or community [16, 17] are identified as significant contributors to the management of chronic illnesses. Table 2 lists the interventions provided to participants in this group.

#### Intervention implementation: cognitive

Cognitive interventions are proposed wherein cognitions are modified to aid in attaining better mental health. The emotional and psychological well-being of a patient is vital for the management of chronic illnesses [18]. Cognitive realisation for the need and importance of adhering to medication is necessary. Cognitive interventions will include (1) video interventions highlighting risk factors associated with various chronic conditions, symptoms of anxiety and depression, and cognitive and behavioural steps to manage anxiety and depression and (2) strategies to improve social cohesion and communication, such as encouraging common meals, family routines, etc. Patients with severe cognitive dysfunction will be referred to the nearest family counselling centre (FCC).

##### Personnel involved

ASHA workers will initiate the implementation because they already have an established trusting relationship with participants and can serve as an essential link to reach out to the community. During the intervention phase, ASHA workers will meet study participants once in a month to assess the compliance to the intervention programme and participation. Other personnel include NGOs, community clubs, youth volunteers, and FCCs funded by the central social welfare board. Monitoring and coordination will be conducted by the research team and CHSWs. Two-day training will be provided to all ASHA workers prior to the implementation of the interventions.

##### Materials for intervention

Dietary charts, health calendars, brochures, and pamphlets constitute a significant part of the intervention. In addition, a mobile application developed with the help of a software developer will be a main part of the assessment and categorisation of participants.

### Sample size

In the community, 36.33% people aged more than 30 years were found to have HTN and/or DM [9]. Thus, this cluster RCT will include 1452 participants and their families (726 patients in each arm). Trained and certified project staff will collect baseline data by using study questionnaires. Project staff, under the supervision of the principal investigator, will complete data entry into computer systems. The data will be collated, cleaned, and analysed centrally. Furthermore, the accuracy of data entry in randomly selected fields will be independently checked by the principal investigator of the study.

### Statistical analysis

Data will be analysed on the basis of the intention to treat principle. Changes observed from the baseline and at follow-up will be compared between the intervention and usual care groups. All data will be de-identified using a unique ID to ensure the confidentiality of information. Upon implementation of the intervention, follow-up measurements will be taken at 6 and 12 months from the baseline. Data will be generated from standardised questionnaires and assessment procedures. Baseline characteristics will be described using means (with standard deviations) or frequencies based on the type of variable. The mean difference between the groups will be examined to understand the effects of the intervention. Mixed effect analysis of covariance will be employed to test for interactions between time (pre and post) and group (intervention or usual care), and partial eta squared (ηp^2^) will be reported as a measure of effect size. The comparisons of variables will be adjusted for potential confounders. The intention-to-treat analysis will be used to observe changes occurred throughout the year during follow-up in participants assigned to the Swāsthya intervention and usual care. The effect attributable to the intervention will be estimated by calculating mean differences between the groups at each of the follow-up points (at the baseline and at 6 and 12 months after the baseline; adjusted odds ratios for dichotomous outcome variables at 95% confidence intervals). Precautions will be taken to rule out the potential interaction effects between the Swāsthya and usual care groups. Software-assisted multiple imputation methods up to five times will be used to deal with missing values. All statistical analyses will be performed using STATA-14 and R software. The effectiveness of the intervention model will be ascertained 1 year after the completion of the study, and the model in itself will also be made public. Information on the study will be available on the website of the college as well as reported in scientific journals and at scientific conferences.

### Ethical oversight

Ethical approval is obtained from the Institutional Review Board of Rajagiri College of Social Sciences (RIRB2020009) and is registered under Clinical Trial Registry-India. The modified intervention programme will be piloted to test its feasibility and will be pretested for its validity and reliability. It will further be modified on the basis of insights from these processes. For the intervention programme, participants will be informed regarding the study purpose and provided with a detailed information sheet. Community volunteers will obtain written informed consent from all study participants. All personal information will be kept confidential. To ensure confidentiality and anonymity, participants will be given unique numbers while entering data into the system. All changes in the trial protocol will be informed to the institutional review board.

## Discussion

Targeted prevention and management of chronic conditions are effective strategies that can reduce the public health burden imposed by NCDs in our country. Involving family members and engaging ASHA workers and other community members in health promotion can allow for the sustainability of interventions by harnessing the potential of existing healthcare resources. Furthermore, the delivery of lifestyle interventions at the family level, tapping the collectivistic nature of the Indian society that is built on cooperation and mutual interdependence, is crucial. Through task-sharing and task-shifting strategies, ASHA workers, during their routine visits, can effectively and positively interact and educate households to make healthy changes within the whole family, equip them with opportunities to make behavioural and lifestyle changes, and initiate strategies to mobilise social support and communication and reduce mental health issues. Recruiting CHSWs to navigate the community-based NCD management regimen to ensure quality service is the innovation of this project. This is important because although ASHA workers are accepted into the community, they may not have the competency to deal with the complex biopsychosocial determinants of chronic illness management. Utilising their knowledge, competencies, managerial skills, and humanistic and democratic values, CHSWs will be able to navigate formal and informal services, mobilising social and economic resources through geographical and functional coordination. All health intervention materials will be aided by simple yet powerful brochures and pamphlets with the pictorial representation of key messages.

The outcome of the proposed programme would be a reduction in behavioural, social, and cognitive risk factors for chronic conditions in participants and increased treatment adherence and medication compliance with an overall aim of controlling the incidence of chronic conditions in the society. A multidimensional, family-based chronic condition risk management programme navigated by CHSWs through care coordination strategies has not been implemented before in the country. Our study will also provide evidence to support the role of care coordination in the task shifting and task sharing of the existing community health workforce in the prevention and management of HTN and DM in India. Knowledge generated from this trial can significantly contribute to policy changes and help develop improved guidelines for clinical practice, leading to a reduction in the incidence of NCDs in India and other LMICs. Most importantly, the results of this study will facilitate a paradigm shift in the management of chronic conditions as well as a shift from a medical model to a multidimensional model focusing on the biopsychosocial aspect of the control of chronic conditions.

### Trial status

This intervention trial is registered under Clinical Trial Registry India (ICMR-NIMS) on 1st December 2020; with the registration number (CTRI/2020/12/029474). Participant recruitment will start on 1st February 2021 and the whole process is likely to be completed by 31st December 2021.Trial is publically available on the following link: http://ctri.nic.in/Clinicaltrials/pmaindet2.php?trialid=46299&EncHid=&userName=An%20Integrated%20Chronic%20Condition%20Management.

## Data Availability

Not Applicable. Data generated after participant recruitment will be made available upon reasonable request from the corresponding author once the preliminary results are published.

## Abbreviations

NCD: Non communicable diseases
DM: Diabetes mellitus
HTN: Hypertension
CC: Chronic conditions
PHC: Primary Health Centre
ASHA: Accredited Social Health Activist
BMI: Body mass index
BP: Blood pressure
DMO: District Medical Office
NGO: Non governmental organizations
FCC: Family counselling centres
